# Prevalence of autoimmune thyroid diseases among the Turner Syndrome patients: meta-analysis of cross sectional studies

**DOI:** 10.1186/s13104-018-3950-0

**Published:** 2018-11-29

**Authors:** Sagad Omer Obeid Mohamed, Ibrahim Hassan Eldaw Elkhidir, Abdelhamid Ibrahim Hassan Abuzied, Ahmed Abdulgadir Mohammed Hassan Noureddin, Gehad Abdelmonem Abdalla Ibrahim, Ahmed Abdallah Ali Mahmoud

**Affiliations:** Faculty of Medicine, University of Khartoum, Qasr Street, P.O. Box 11111, Khartoum, Sudan

**Keywords:** Thyroid diseases, Turner Syndrome, Prevalence, Meta-analysis

## Abstract

**Objective:**

This meta-analysis was done to estimate the prevalence of autoimmune thyroid diseases (ATDs) in Turner Syndrome patients, and to determine the clinical status of thyroid autoimmune diseases that occur frequently in association with Turner Syndrome.

**Results:**

A total of 18 studies were included in the meta-analysis. The pooled overall prevalence of autoimmune thyroid diseases in Turner Syndrome patients was 38.6% (95% CI 29.7–47.6%), with 12.7% (95% CI 9.30–16.1%) of them had clinical hypothyroidism and 2.6% (95% CI 1.5–3.8%) had hyperthyroidism. I-squared test had a high result of heterogeneity. In subgroup analyses, the prevalence of ATDs was higher in the European region than Asian region. Autoimmune thyroid diseases are commonly associated with Turner Syndrome. Early detection of thyroid diseases by optimal screening among children with Turner Syndrome is required to ensure effective management.

**Electronic supplementary material:**

The online version of this article (10.1186/s13104-018-3950-0) contains supplementary material, which is available to authorized users.

## Introduction

Turner Syndrome (TS) is one of the most common chromosomopathies caused by numeric or structural abnormalities of the X chromosome [1–3]. It has a prevalence of 25–210 per 100,000 females [1]. Frequent clinical features include short stature, webbed neck, broad chest, cubitus valgus, delayed puberty and gonadal dysgenesis [4, 5].

Morbidity is considerably increased in Turner Syndrome. Several autoimmune disorders are more frequent in girls with Turner Syndrome such as: autoimmune thyroid diseases (ATDs), celiac disease, inflammatory bowel disease, psoriasis, vitiligo and juvenile rheumatoid arthritis. Likewise, there is increased risk of ischemic heart disease, hypertension and congenital malformations of the heart and urinary system [1–6].

Previous studies identified that haploinsufficiency of at least ten genes located on the X chromosome are involved in the immune regulation process, affecting self-protein exposure in the thymus and escape of auto-reactive T cells [3, 7, 8]. Bakalov et al. findings suggest that factors associated with ovarian insufficiency may be responsible for this autoimmune condition [9].

ATDs had been reported to be more frequent in girls with TS than in the general population [6, 10, 11], covering a spectrum of phenotypes. They include Hashimoto’s thyroiditis (HT)—which is considered the most common autoimmune thyroid disease, and Graves’ disease (GD)—which causes hyperthyroidism [7, 12–16]. Most HT forms evolve into hypothyroidism, although at presentation patients can be without clinical hypothyroidism [16].

Previous studies of the magnitude of ATDs among Turner Syndrome population remains inconsistent; wide variation of percentages for the presence of ATDs occurs in the studies [7]. Furthermore, to the best of our knowledge, there is no meta-analysis of existing contemporary evidence on the prevalence of ATDs among the TS population. Therefore, the aim of this meta-analysis is to estimate the prevalence and clinical status of ATDs in patients with TS.

## Main text

### Methods

In August 2018, a literature search using the electronic databases of MEDLINE/PubMed, ScienceDirect, Google Scholar and OpenGrey was done to identify all of the relevant studies clarifying the prevalence of autoimmune thyroid disease in patients with Turner Syndrome published up to date without time limitation. The search strategy was formulated by using the key words ‘Turner Syndrome’ and ‘thyroid’. Manual search for additional studies was performed using references cited in original study articles. We followed the PRISMA guidelines (Preferred Reporting Items for Systematic Reviews and Meta-Analyses) [17].

Cross-sectional studies published in English, with sufficient information to estimate the prevalence of autoimmune thyroid diseases in patients with Turner Syndrome were included in this study. Study exclusion criteria included the following: Case reports, letters, editorials and studies lacking the relevant data or reported only the odd ratio in follow-up years were excluded.

Two reviewers (Sagad and Ibrahim) screened the titles and abstracts of the identified studies and assessed the full text of potentially eligible studies. Any disparity was resolved by consensus. The following information extracted from each article: authors, year, region of study, sample size and reported overall prevalence of thyroid autoimmune diseases and clinical status of thyroid diseases. We extracted the relevant data using Microsoft Office Excel.

The included studies were assessed for quality; two authors (Sagad, Ibrahim) independently using Newcastle–Ottawa Scale adapted for cross sectional studies assessment tool, with a score of ≥ 5 out of 10 considered as high quality score. We included studies of high quality score in this meta-analysis.

Relevant data were exported to OpenMeta Analyst version 10.10 software for analysis [18]. Meta-analysis of pooled prevalence with 95% CIs was carried out using a random effects model, and results were displayed in a forest plot. Heterogeneity among studies was estimated using the Cochran’s Q and I^2^ statistic and is considered as low, moderate or high for 25%, 50%, and 75%, respectively. Publication bias was determined using Comprehensive Meta Analysis software based on Begg’s test, Egger’s test and the symmetry of funnel plot [19, 20], and Duvall and Tweedie trim and fill method was considered for final effect size estimation when publication bias detected [21]. Subgroup analyses by continents were carried out due to high heterogeneity between studies. Significance level was set at 0.05.

### Results

After reviewing titles and abstracts of the identified studies, 704 studies were excluded. Full texts of 33 studies were screened and 15 studies of which were subsequently omitted because of low quality and insufficient data to estimate the outcomes of interest. A total of 18 studies published from (1986) to (2018) representing 2719 patients which fulfilled the eligibility criteria, were included in this meta-analysis (Table 1). The process of study selection is summarized in a flow chart shown in Additional file 1: Fig S1. Ten of the included studies were done in Europe, five studies from Asia, two from USA and one from Egypt.

**Table 1:** The descriptive summary of 18 studies on the prevalence of thyroid autoimmune diseases among patients with Turner Syndrome

Authors	Year	Country	Sample size	No. of patients with HT	No. of patients with clinical hypothyroidism	No. of patients with clinical hyperthyroidism	Total no. of patients with ATDs
Hamza et al. [22]	2013	Egypt	80	40	5	2	54
Bakalov et al. [9]	2012	USA	244	82	Na	Na	82
Germain et al. [23]	1986	USA	54	30	12	0	30
Yesilkaya et al. [24]	2015	Turkey	792	79	42	3	82
Grossi et al. [7]	2013	Italy	66	26	14	0	26
Hoxha et al. [25]	2015	Albania	59	39	20	0	39
Bettendorf et al. [26]	2006	Germany	120	43	24	0	43
Calcaterra et al. [27]	2011	Italy	73	32	Na	Na	32
Mortensen et al. [28]	2009	Denmark	107	59	16	2	61
Elsheikh et al. [29]	2001	UK	145	59	14	1	60
Radetti et al. [30]	1995	Italy	478	103	29	3	106
Kerdanet et al. [31]	1994	France	67	14	Na	Na	14
Livadas et al. [32]	2005	Greece	84	49	18	2	51
Pin Chang et al. [33]	2000	Taiwan	77	19	1	2	21
Chen et al. [34]	2015	China	69	20	4	3	23
Fukuda et al. [35]	2009	Japan	61	34	20	3	37
Huang et al. [36]	2018	Taiwan	19	1	1	1	2
Wu. et al. [37]	2017	China	124	17	17	1	18

*No.* number, *Na* not available, *ATD* autoimmune thyroid disease, *HT* Hashimoto’s thyroiditis

**Fig. 1:**
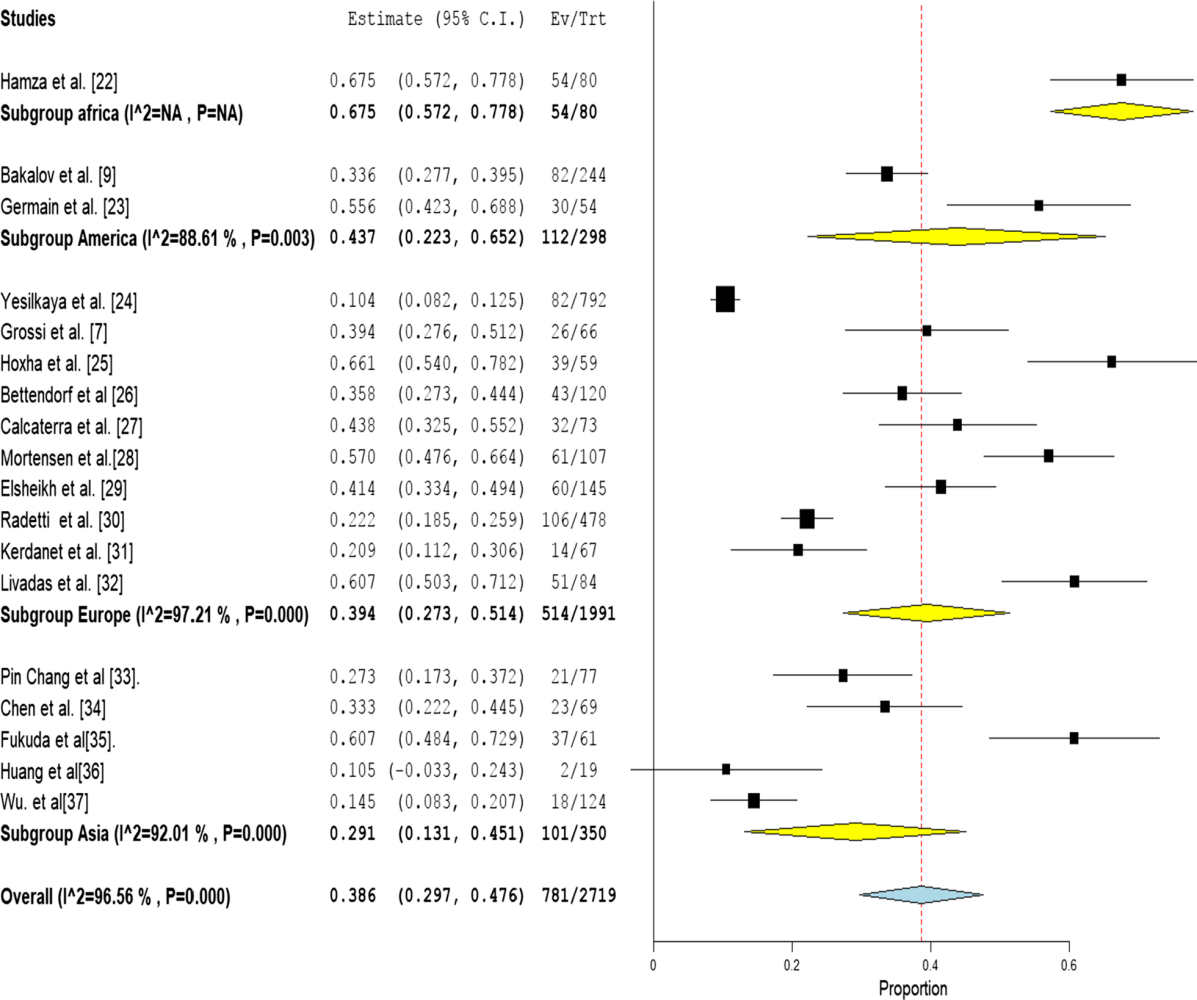
Pooled prevalence of autoimmune thyroid diseases among patients with Turner Syndrome

Prevalence of autoimmune thyroid diseases ranged from 10.5 to 67.5% among reviewed studies. However, meta-analysis for the included studies showed that the pooled prevalence of ATDs among TS population was 38.6% (95% CI 29.7–47.6%) (Fig. 1), I^2^ test showed a high level of heterogeneity (I^2^ = 96.6%, P < 0.001). Based on subgroup analyses by continents, the pooled prevalence of ATDs among patients was 39.4% (95 CI 27.3–51.4%) in Europe, and 29.1% (95% CI 13.3–45.1%) in Asia (Fig. 1). Publication bias was detected based on Begg’s test (P < 0.02) and asymmetry of funnel plot (Additional file 2: Fig S2). Duvall and Tweedie trim and fill method indicated (6) potential studies missing, and the point estimate was 25.5% (95% CI 17.8–35%).

Pooled estimate showed that 34.2% (95% CI 26.7–41.6%) of the patients had HT (I^2^ = 94.9%, P < 0.001) (Additional file 3: Fig S3), 12.7% (95% CI 9.30–16.1%) of the patients had clinical hypothyroidism (I^2^ = 87.47%, P < 0.001) (Additional file 4: Fig S4), and 2.6% (95% CI 1.5–3.8%) had hyperthyroidism (I^2^ = 0%, P = 0.777) (Additional file 5: Fig S5).

We included 10 studies which assessed the association of karyotyping with the occurrence of ATDs among the Turner Syndrome patients. Frequency of ATDs was higher in patients with Iso-Xq karyotype (66.2%) (95% CI 54.4–78.0%) (Additional file 6: Fig S6) than patients with monosomy 45, X (36.9%) (95% CI 27.8–46.0%) (Additional file 7: Fig S7) and other karyotypes (33.5%) (95% CI 22.9–44.1%) (Additional file 8: Fig S8) (Table 2).

**Table 2:** Descriptive summary of 10 studies on the prevalence of ATDs among patients with different karyotypes of Turner Syndrome

Authors	No. of patients (monosomy X)	No. of patients with ATD (monosomy X)	No. of patients (iso-Xq)	No. of patients with ATD (iso-Xq)	No. of patients (other karyotypes)	No. of patients with ATD (other karyotypes)
Hamza et al. [22]	37	14	19	16	24	10
Grossi et al. [7]	31	14	6	5	29	7
Bettendorf et al. [26]	72	30	7	4	40	12
Mortensen et al. [28]	64	31	23	9	20	8
Elsheikh et al. [29]	86	35	24	20	35	5
Kerdanet et al. [31]	25	2	16	11	31	5
Livadas et al. [32]	22	10	7	5	22	9
Pin Chang et al. [33]	36	10	4	1	20	10
Chen et al. [34]	28	10	12	7	29	6
Fukuda et al. [35]	20	9	20	13	21	15

*No.* number, *Na* not available, *ATD* autoimmune thyroid disease

### Discussion

We have updated estimate of the global prevalence of autoimmune thyroid diseases in Turner Syndrome population. Existing evidence from 18 included studies showed that more than one-third of patients with Turner Syndrome had autoimmune thyroid diseases. This finding is higher as compared to previous reviews done by Gravholt and Davenport [38, 39] reporting 10–25% as prevalence of autoimmune thyroid disease among Turner Syndrome population. Likewise, De Marqui et al. and Chen et al. [5, 34] revealed that ATDs are the most common autoimmune disorders occur in TS.

Discrepancy between prevalence rates from included studies might be attributable to the differences of karyotyping, diagnostic methods and age of presentation among patients [7], as well as demographic characteristics [34]. In this meta-analysis, the prevalence rate of ATDs in iso-Xq karyotype patient was higher than other karyotypes. Effect of karyotyping was supported by several studies. Bakalov et al. and Elsheikh et al. [9, 29] reported that the risk for HT was higher in the Iso-chromosome Xq population. Mortensen et al. and Chen et al. [28, 34] showed that the frequency of ATDs increase with age, although ATDs can occur before the age of 8 years [7]. Previous systematic review suggests screening for thyroid diseases to continue throughout adult life [11].

Findings of this meta-analysis confirm higher prevalence rate of hypothyroidism status than grave’s disease in TS patients diagnosed with thyroid autoimmunity. Similar findings were reported by several studies [29, 30, 40, 41], and Hashimoto’s thyroiditis was found to be the most common autoimmune disorder in TS girls [40]. Euthyroidism with absence of signs and symptoms a common clinical phenotype in TS patients diagnosed with thyroid autoimmunity. Transition from one clinical phenotype to another over time can occur [14].

Geographic gaps exist in the data on ATDs in Turner Syndrome population, especially in the American and African countries. The studies included in this meta-analysis were mostly from Europe and only one study from Africa.

Autoimmune thyroid diseases occur with increased frequency in patients with Turner Syndrome. Health care providers need to be aware of the clinically important association between thyroid diseases and Turner Syndrome. Optimal screening and early detection of thyroid diseases among children with Turner Syndrome are required to ensure effective management.

## Limitations

The findings of this study need to be considered in the context of some limitations. Articles in non-indexed journals and non-published papers were not searched, which might make publication bias. Inclusion of studies published only in English may cause language bias. Also, this study did not determine the possible risk factors that may contribute for the occurrence of ATDs in Turner Syndrome patients.

## Additional files

**Additional file 1: Fig S1.** The flow diagram for the process of study selection and systematic review of literature.

**Additional file 2: Fig S2.** Funnel plot showing evidence of publication bias among 18 studies in a meta-analysis of ATDs prevalence in TS population. Six missing studies added in the left side.

**Additional file 3: Fig S3.** Pooled prevalence of HT among patients with Turner Syndrome diagnosed with ATDs.

**Additional file 4: Fig S4.** Pooled prevalence of clinical hypothyroidism among patients with Turner Syndrome diagnosed with ATDs.

**Additional file 5: Fig S5.** Pooled prevalence of hyperthyroidism among patients with Turner Syndrome diagnosed with ATDs.

**Additional file 6: Fig S6.** Pooled prevalence of ATDs among patients with Iso-Xq karyotype of Turner Syndrome.

**Additional file 7: Fig S7** Pooled prevalence of ATDs among patients with monosomy 45, X karyotype of Turner Syndrome.

**Additional file 8: Fig S8.** Pooled prevalence of ATDs among patients with other forms of karyotypes of Turner Syndrome.

## Abbreviations

ATD: autoimmune thyroid disease
TS: Turner Syndrome
HT: Hashimoto’s thyroiditis
GD: Graves’ disease
