# Supplementary material for: Prevalence of autoimmune thyroid diseases among the Turner Syndrome patients: meta-analysis of cross sectional studies
Source: BMC Res Notes. 2018 Nov 29;11:842. doi: 10.1186/s13104-018-3950-0 (PMC6264051; doi:10.1186/s13104-018-3950-0)
Supplement: Supplementary file 1 — Additional file 1: Fig S1. The flow diagram for the process of study selection and systematic review of literature. [file 13104_2018_3950_MOESM1_ESM.docx]

Records excluded
(n = 707 )

## Identification

## Eligibility

## Included

## Screening

Records identified through database searching
(n =732 )

Additional records identified through other sources (reference lists of the articles)
(n = 8 )

Records screened

(n = 740 )

Full-text articles assessed for eligibility
(n = 33 )

Full-text articles excluded, with reasons
(n = 15 )

Studies included in qualitative synthesis
(n = 18 )

Studies included in quantitative synthesis (meta-analysis)
(n = 18 )
