# Supplementary figures and images for: Prevalence of autoimmune thyroid diseases among the Turner Syndrome patients: meta-analysis of cross sectional studies
Source: BMC Res Notes. 2018 Nov 29;11:842. doi: 10.1186/s13104-018-3950-0 (PMC6264051; doi:10.1186/s13104-018-3950-0)

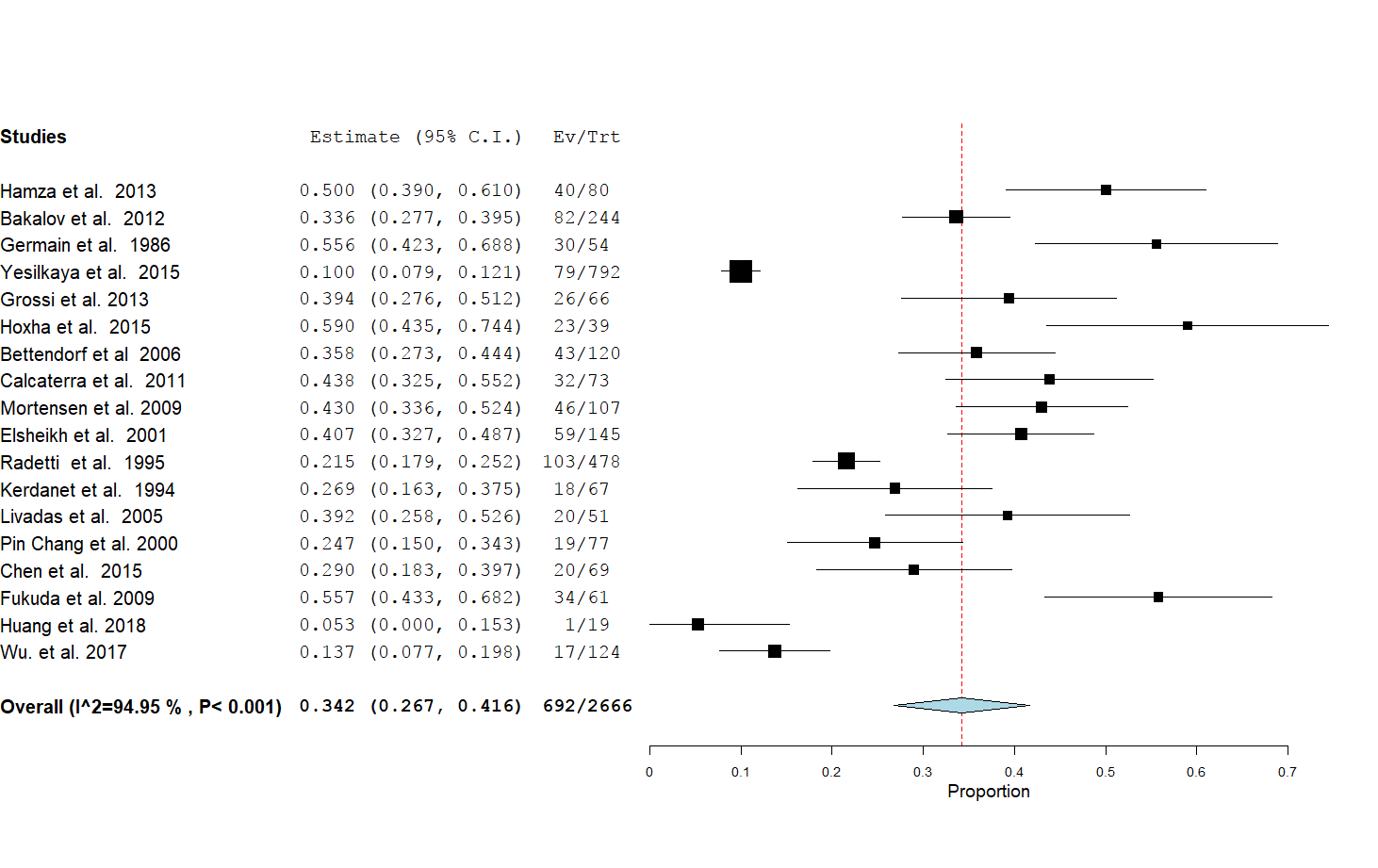

Supplement: Supplementary file 3 — Additional file 3: Fig S3. Pooled prevalence of HT among patients with Turner Syndrome diagnosed with ATDs. [file 13104_2018_3950_MOESM3_ESM.docx]

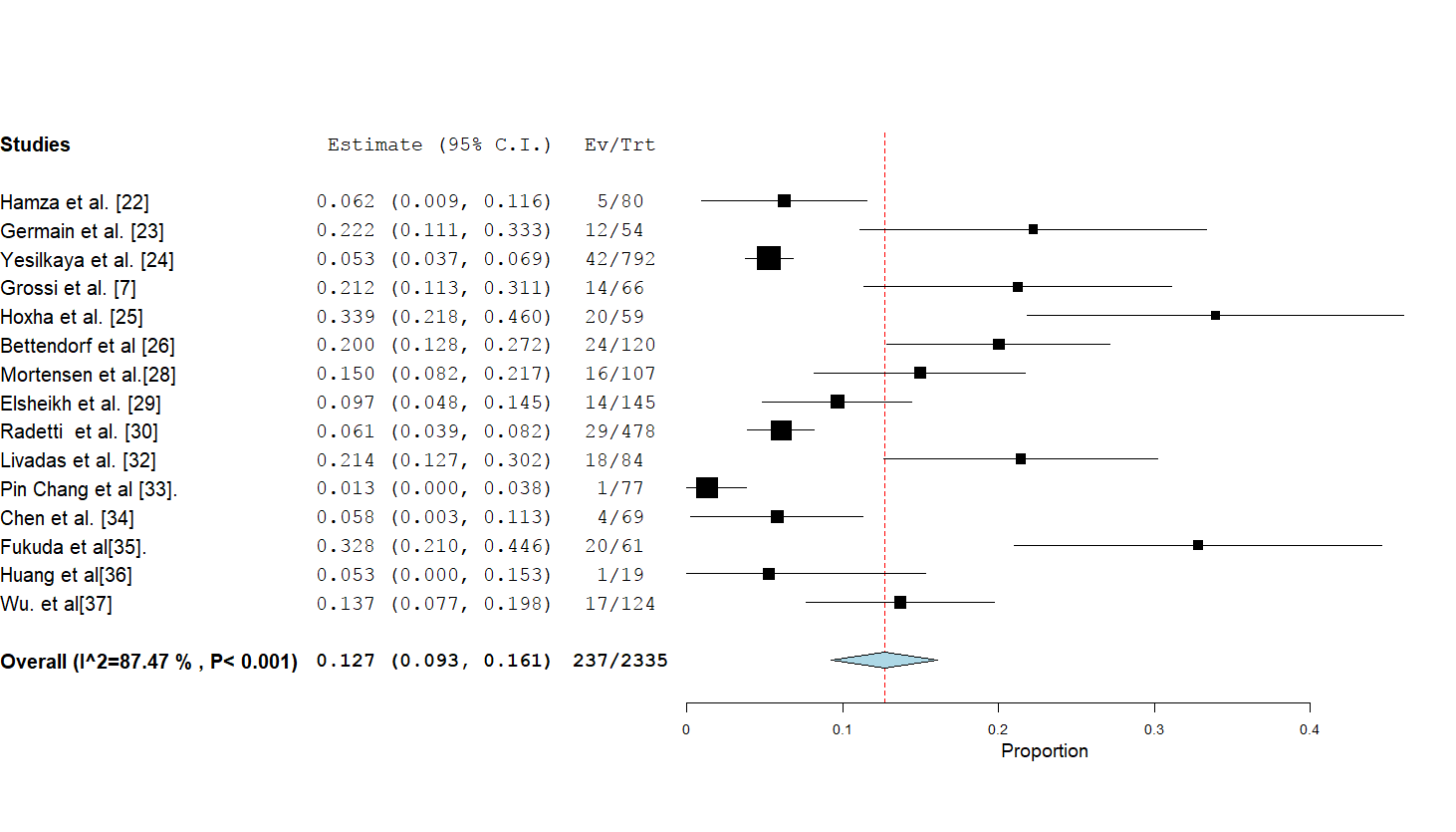

Supplement: Supplementary file 4 — Additional file 4: Fig S4. Pooled prevalence of clinical hypothyroidism among patients with Turner Syndrome diagnosed with ATDs. [file 13104_2018_3950_MOESM4_ESM.docx]

**
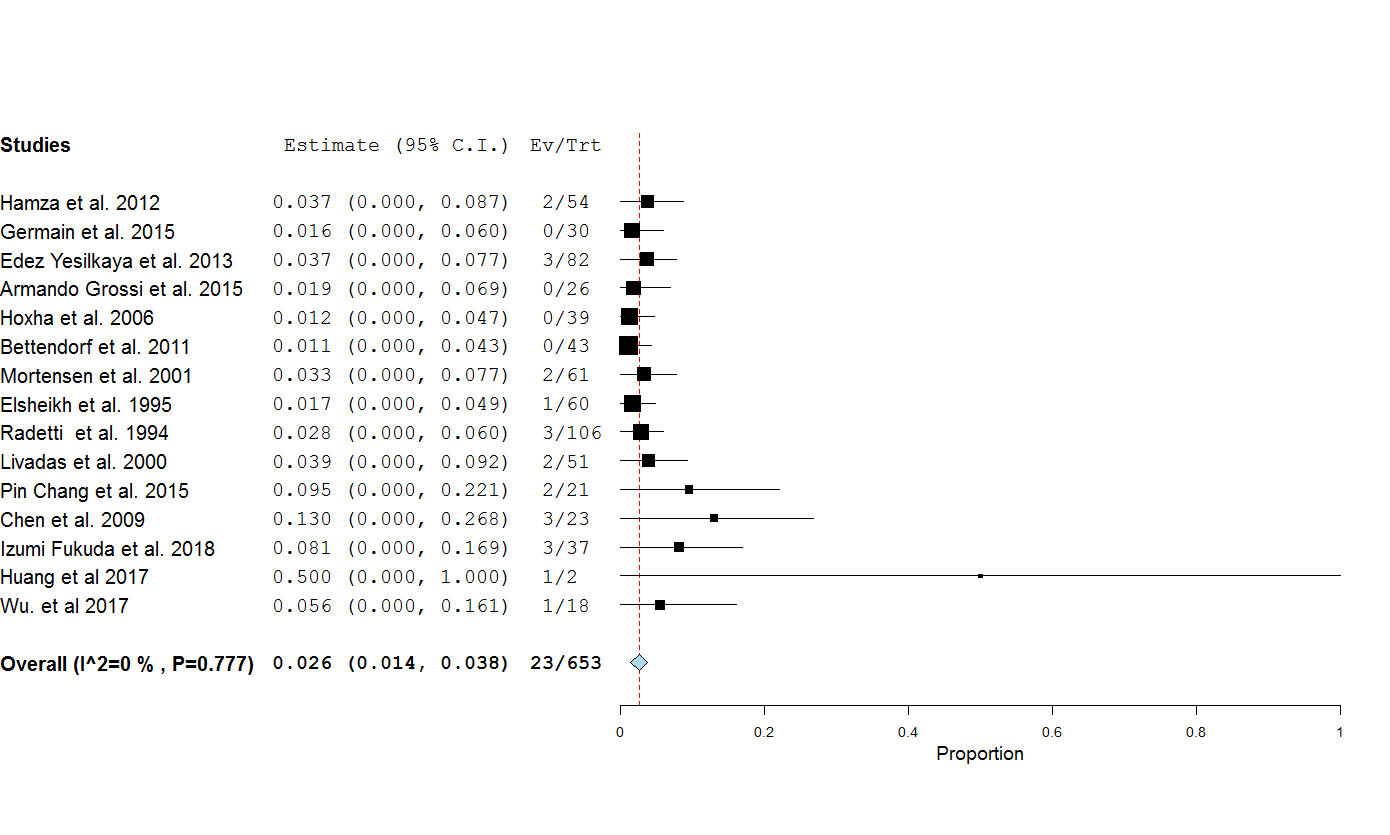
**

Supplement: Supplementary file 5 — Additional file 5: Fig S5. Pooled prevalence of hyperthyroidism among patients with Turner Syndrome diagnosed with ATDs. [file 13104_2018_3950_MOESM5_ESM.docx]

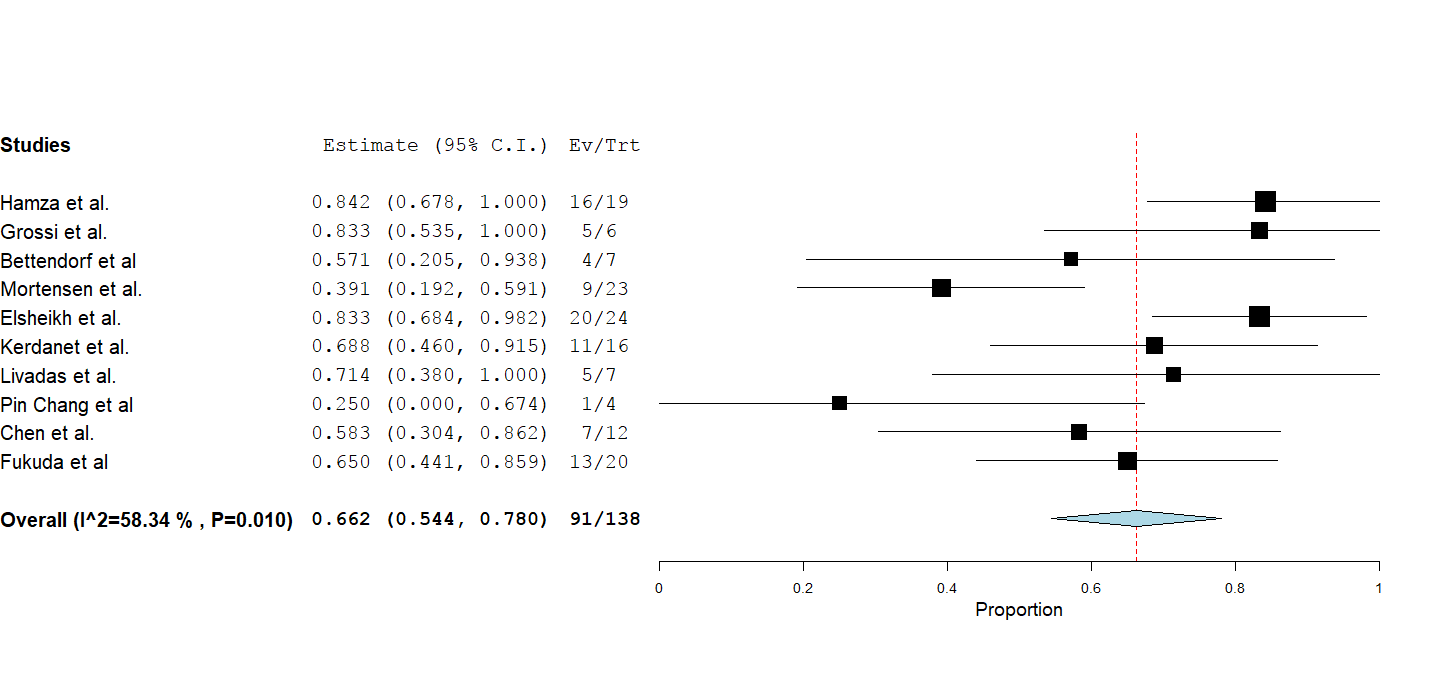

Supplement: Supplementary file 6 — Additional file 6: Fig S6. Pooled prevalence of ATDs among patients with Iso-Xq karyotype of Turner Syndrome. [file 13104_2018_3950_MOESM6_ESM.docx]

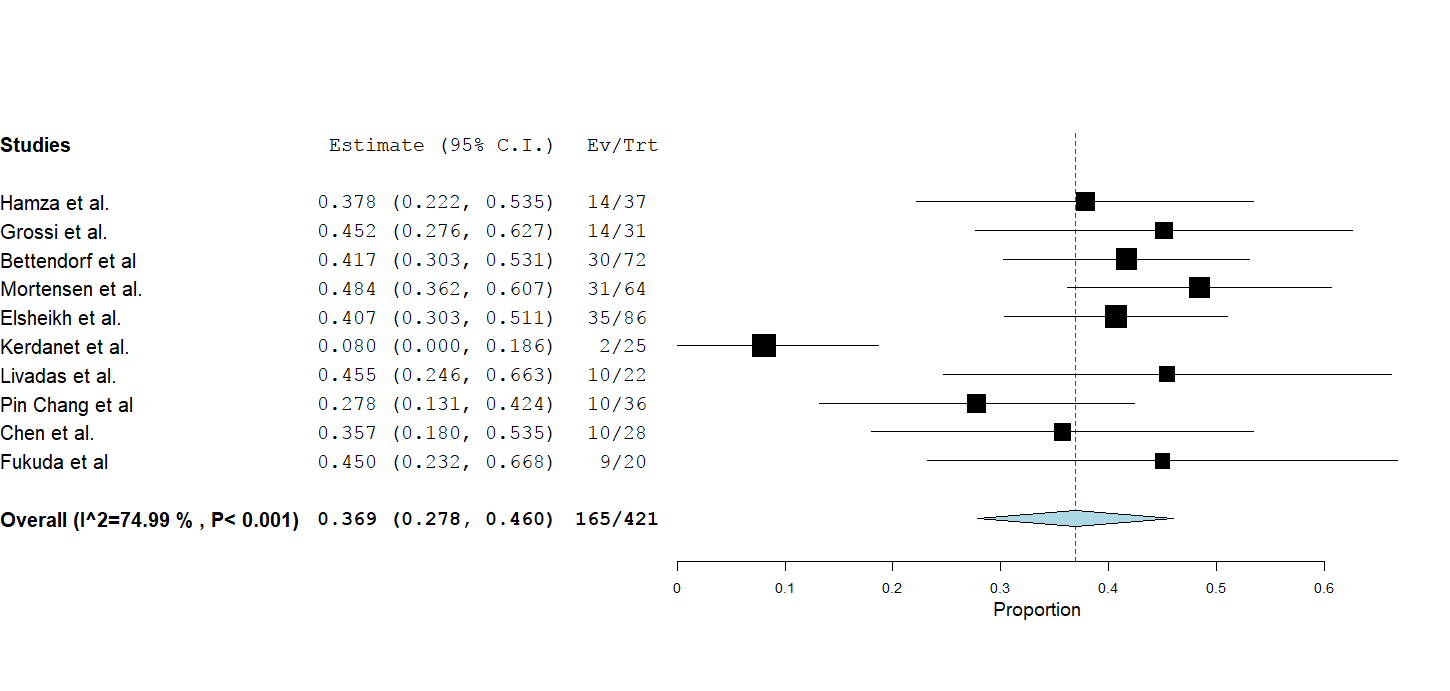

Supplement: Supplementary file 7 — Additional file 7: Fig S7 Pooled prevalence of ATDs among patients with monosomy 45, X karyotype of Turner Syndrome. [file 13104_2018_3950_MOESM7_ESM.docx]

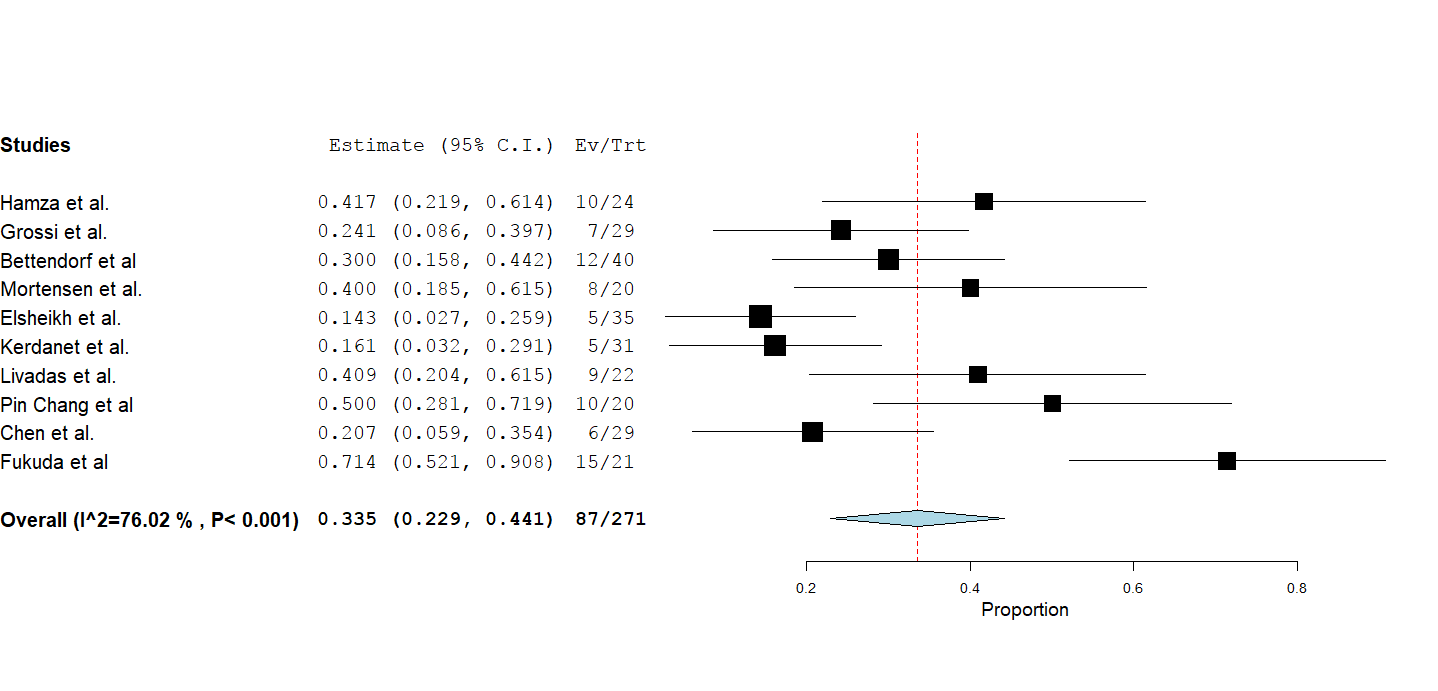

Supplement: Supplementary file 8 — Additional file 8: Fig S8. Pooled prevalence of ATDs among patients with other forms of karyotypes of Turner Syndrome. [file 13104_2018_3950_MOESM8_ESM.docx]
