# Supplementary material for: Prevalence of autoimmune thyroid diseases among the Turner Syndrome patients: meta-analysis of cross sectional studies
Source: BMC Res Notes. 2018 Nov 29;11:842. doi: 10.1186/s13104-018-3950-0 (PMC6264051; doi:10.1186/s13104-018-3950-0)
Supplement: Supplementary file 3 [file 13104_2018_3950_MOESM2_ESM.docx]

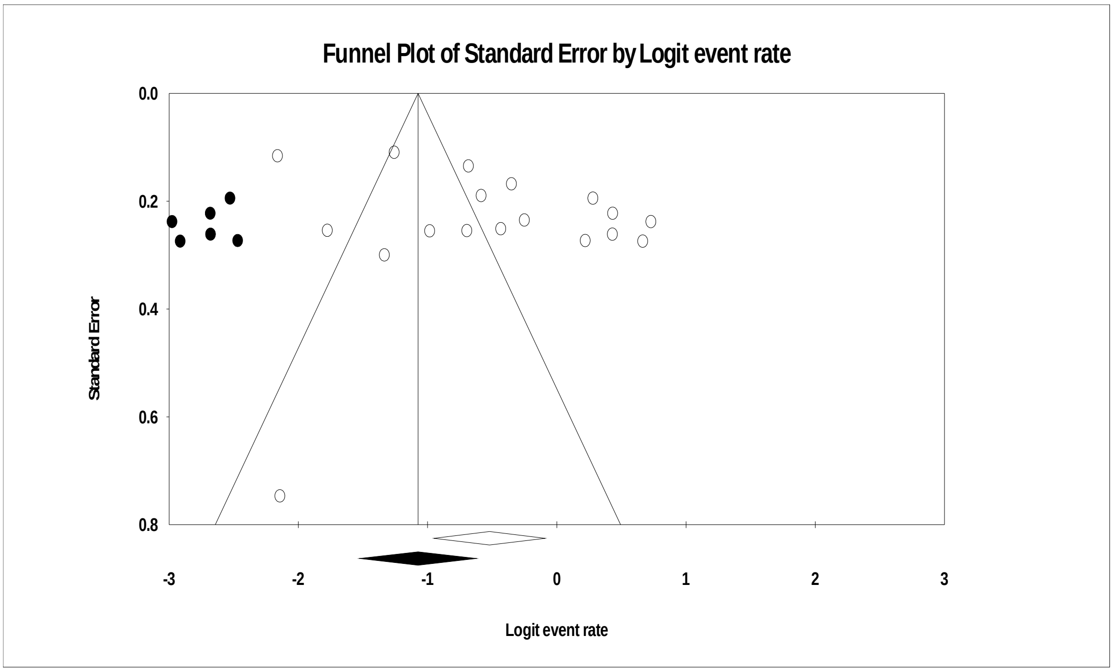

Funnel Plot of Standard Error by Logit event rate
Standard Error
0.0
0.2
0.4
0.6
0.8
-3
-2
-1
0
1
2
3
Logit event rate
